# Embryogenic Callus as Target for Efficient Transformation of *Cyclamen persicum* Enabling Gene Function Studies

**DOI:** 10.3389/fpls.2018.01035

**Published:** 2018-07-24

**Authors:** Svenja Ratjens, Samuel Mortensen, Antje Kumpf, Melanie Bartsch, Traud Winkelmann

**Affiliations:** Institute of Horticultural Production Systems, Leibniz Universität Hannover, Hanover, Germany

**Keywords:** *Agrobacterium tumefaciens*, auxin, DR5 promoter, embryogenic callus, ornamental plant, redox sensor roGFP2_Orp1, somatic embryogenesis

## Abstract

*Cyclamen persicum* is an ornamental plant with economic relevance in many parts of the world. Moreover, it can be regarded as an applied model for somatic embryogenesis, since transcriptomic, proteomic, and metabolomic comparisons have revealed insights into this regeneration process on the molecular level. To enable gene function analyses, the aim of this study was to establish an efficient *Agrobacterium tumefaciens*-mediated genetic transformation protocol for *C. persicum*. For the first time, embryogenic callus cultures were used as a target material. The advantages of embryogenic callus are the defined and known genotype compared to seedlings, the high regeneration potential and the stability of the regenerated plants. *A. tumefaciens* strains EHA105 and LBA4404 were most efficient for transformation, resulting in transformation efficiencies of up to 43 and 20%, respectively. In regenerated plants, the presence of the transgenes was verified by PCR, Southern hybridization, and a histochemical GUS assay. The protocol was applied successfully to two *C. persicum* genotypes. Moreover, it served to transfer two reporter constructs, the auxin-responsive promoter DR5 driving the *gus* gene and the redox sensor roGFP2_Orp1, to the *C. persicum* genotypes, allowing the localization of high auxin concentrations and reactive oxygen species in order to study their roles in somatic embryogenesis in the future. For success in transformation, we regard the following factors as important: highly embryogenic cell lines, the use of Silwet^®^ L-77 as a surfactant during co-culture, a genotype-specific appropriate selection schedule with hygromycin, and *A. tumefaciens* strains EHA105 and LBA4404.

## Introduction

Cyclamen (*Cyclamen persicum* Mill.) is an ornamental crop of high economic relevance in temperate regions, mainly of Europe and Japan (Schwenkel, 2001). Commercially, it is propagated via seeds, but interest exists in vegetative propagation due to the costs of manual emasculation and pollination, and despite F_1_ hybrid breeding programs, the remaining inhomogeneity in some cultivars. Clonal propagation for multiplication of the parental genotypes of F_1_ hybrid cultivars and for mass propagation of selected superior plants can only be achieved by employing *in vitro* culture techniques. Among these, somatic embryogenesis in particular was proposed to be an efficient method of vegetative propagation and has been introduced for cyclamen by several groups (e.g., Wicart et al., 1984; Otani and Shimada, 1991; Kiviharju et al., 1992; Kreuger et al., 1995; Takamura et al., 1995; Schwenkel and Winkelmann, 1998; Winkelmann, 2010, reviewed by Jalali et al., 2012). Although this technique is in principle applicable to a wide range of genotypes (Winkelmann and Serek, 2005; Winkelmann, 2010), different genotype-dependent efficiencies and problems such as asynchronous development (Rode et al., 2011), precocious germination, lack of desiccation tolerance, or the absence of a growth arrest connected to maturation (Schmidt et al., 2006) have been assigned to somatic embryogenesis in cyclamen. Thus, a better understanding of the molecular and physiological control of the regeneration pathway is needed. Insights have been gained from comparisons of somatic and zygotic embryos on the transcriptomic (Hoenemann et al., 2010), proteomic (Winkelmann et al., 2006a; Bian et al., 2010; Rode et al., 2011; Mwangi et al., 2013), and metabolomic (Winkelmann et al., 2015) levels. These analyses have shown that somatic embryos are less well protected from stress than zygotic embryos are and that they lack seed storage proteins.

The key prerequisite for testing the candidate genes identified in the abovementioned comparative approaches for their functional roles in somatic embryogenesis is an efficient transformation protocol allowing overexpression, knock-down via RNA interference and knock-out using CRISPR-Cas approaches. Genetic transformation of cyclamen via *Agrobacterium tumefaciens* was first reported by Aida et al. (1999). Since then, only a very few reports of *A. tumefaciens*-mediated transformation have been published (**Table 1**), most of them based on adventitious shoot formation from etiolated seedlings. Seedlings, however, have the disadvantage of unknown genetic constitution in this allogamous species. When comparing the regeneration pathways organogenesis (adventitious shoot formation) and somatic embryogenesis in *C. persicum*, somatic embryogenesis stands out due to the high number of regenerated plants and their true-to-typeness in many genotypes (Schwenkel and Winkelmann, 1998). Moreover, single or only few cells give rise to the formation of somatic embryos, thus reducing the risk of chimeras. Additionally, due to the easy scalability and the less hands-on time, embryogenic cultures are a highly suitable target material for *A. tumefaciens-*mediated transformation allowing genetic studies in a clonal offspring.

**Table 1:** Overview of published reports on genetic transformation of *Cyclamen persicum*.

Regeneration pathway	Target material (explants)	Pre-culture	*Agrobacterium tumefaciens* strain	Transferred genes	Selection	Transformation efficiency	Reference
Adventitious shoot formation	Etiolated petioles	6 days	AGL-0, LBA4404	*gusA*int, *hpt* & *npt*II	5 mgL^−1^ hyg or 100 mgL^−1^ kan	9–19% (AGL-0); 0–2.5% (LBA4404)	Aida et al., 1999
Adventitious shoot formation	Etiolated hypocotyl segments	1–8 days	LBA4404, EHA105	*npt*II, *gusA*int	50 mgL^−1^ kan	15.3% (LBA4404); 2.3% (EHA105)	Boase et al., 2002
Secondary somatic embryogenesis	Somatic embryos	3 months	LBA4404	*gusA*int, *hpt*	5 mgL^−1^ hyg	0.7% hyg^R^ calluses	Terakawa et al., 2008
Adventitious shoot formation	Etiolated hypocotyl segments	1–8 days	EHA105	*CpF3′5′H* *hpt*	5 mgL^−1^ hyg (12 days); 20 mgL^−1^ hyg (65 days); 15 mgL^−1^ hyg (thereafter)	Not indicated	Boase et al., 2010
Adventitious shoot formation	Etiolated petioles regenerated from leaf explants	–	LBA4404	*CpTCP-SRDX, hpt*	5 mgL^−1^ hyg	53%	Tanaka et al., 2011
Adventitious shoot formation	Etiolated petioles of seedlings	6 days	EHA105	*CpFAD7*, *hpt*	10 mgL^−1^ hyg	Not indicated	Kai et al., 2012
Adventitious shoot formation	Etiolated petioles regenerated from leaf explants	–	GV3101, LBA4404	*CpAG1-SRDX, CpAG2-SRDX, hpt*	5 mgL^−1^ hyg	Not indicated	Tanaka et al., 2013
Somatic embryogenesis	Embryogenic callus	10 days	LBA4404, EHA105, GV2260, GV3101, AGL-1	*hpt, gusAint, DR5::gus, roGFP2_Orp1*,	5–10 mgL^−1^ hyg (14 days), 10–20 mgL^−1^ (thereafter) sensitivity depends on genotype	43% (EHA105), 20% (LBA4404)	Ratjens et al., this study

*gusAint, reporter gene gusA containing an intron; hpt, hygromycin (hyg) phosphotransferase gene; nptII, neomycin phosphotransferase gene conferring kanamycin (kan) resistance; CpF3′5′H, flavonoid 3′5′-hydroxylase of Cyclamen persicum; CpFAD7, omega-3 fatty acid desaturase of Cyclamen persicum; CpTCP-SRDX, Cyclamen persicum TCP transcription factor – repressor; CpAG-SRDX, Cyclamen persicum AGAMOUS – repressor; hyg^*R*^, hygromycin-resistant cells growing on hygromycin-containing medium; DR5, synthetic auxin response element; roGFP2_Orp1, redox-sensitive green fluorescent protein biosensor version 2 integrated with a yeast peroxidase gene.*

In the pioneering works of Aida et al. (1999) and Boase et al. (2002), relatively high transformation efficiencies of 19 and 15.3% were obtained, defined as the number of GUS (β-glucuronidase)-positive, blue-stained shoots at six and two and a half months after transformation, respectively. Somatic embryos were the targets of transformation in only one study, and plants were regenerated via secondary somatic embryogenesis; however, this method had a low transformation efficiency of only 0.7% (Terakawa et al., 2008). When comparing hygromycin (5 mgL^−1^) and kanamycin (100 mgL^−1^) for selection of transgenic shoots in cyclamen, Aida et al. (1999) observed clearly higher transformation efficiencies with hygromycin. Consequently, all selection approaches (**Table 1**) in recent studies have used hygromycin as the selective agent, whereas in the early report of Boase et al. (2002), kanamycin was the selective agent. Genes of interest that have been successfully transferred to *C. persicum* include genes that modify flower color (Boase et al., 2010); flower shape (ruffled petals: Tanaka et al., 2011); fatty acid composition, providing heat tolerance (Kai et al., 2012); and floral organs (double flowers: Tanaka et al., 2013, see also **Table 1**).

The objective of this study was to establish an efficient *A. tumefaciens*-mediated transformation protocol for *C. persicum* using embryogenic callus as a target material. This transformation system will provide a base for gene function analyses. Moreover, the first genes of interest were transferred to cyclamen: a *DR5::gus* (Ulmasov et al., 1997a,b) construct to visualize auxin sensitivity and the *roGFP2_Orp1* redox sensor (Gutscher et al., 2009; Schwarzländer et al., 2016) to localize hydrogen peroxide. From subsequent analyses of their expression patterns, we expect insights into the roles of auxin response and reactive oxygen species (ROS) distribution during somatic embryogenesis.

## Materials and Methods

### Plant Material

Two *C. persicum* genotypes representing different genetic backgrounds were included in the study: Genotype 56/2 refers to one plant of the large-flowered commercial F_1_ hybrid cultivar ‘Maxora Light Purple’ (breeder: Varinova BV, Berkel en Rodenrijs, Netherlands), whereas genotype 3145 represents one plant of the mini-type commercial F_1_ hybrid cultivar ‘Zanetto Light Pink’ (breeder: Syngenta Flowers, Enkhuizen, Netherlands).

#### Induction of Embryogenic Callus Cultures

The protocols for somatic embryogenesis in *C. persicum* have been described in detail previously (Schwenkel and Winkelmann, 1998; Winkelmann et al., 1998; Winkelmann, 2010). Embryogenic callus was induced from unpollinated ovules on propagation medium [half-strength MS (Murashige and Skoog, 1962) medium containing 9.05 μM 2,4-dichlorophenoxyacetic acid and 3.94 μM 2iP (6-(γ,γ-dimethylallylamino)purine) and 4 gL^−1^ Gelrite (Duchefa, Haarlem, Netherlands), pH 5.5-5.6] and propagated for 2–3 years by monthly subculture. At each subculture, callus samples were plated on differentiation medium (see section “Regeneration of Plants via Somatic Embryogenesis”) to check the embryogenesis of the line. The lines developing the highest number of embryos were selected for propagation. All cultures were kept at a temperature of 24°C in darkness.

#### Regeneration of Plants via Somatic Embryogenesis

Somatic embryos differentiated within 4 weeks after plating of 200–300 mg embryogenic callus on plant growth regulator-free differentiation medium [half-strength MS medium solidified with 4 gL^−1^ Gelrite (Duchefa), pH 5.5–5.6]. The developing embryos were picked and transferred to fresh differentiation medium of the same composition for germination. After another 4 weeks or when the cotyledons had reached a length of 1–2 cm, the plantlets were transferred to light (16 h photoperiod provided by fluorescent tubes at 30–50 μmolm^−2^s^−1^) and subcultured every 4 to 8 weeks until plants with well-developed tubers and 2–3 leaves could be transferred to greenhouse conditions.

### *Agrobacterium tumefacien* Strains and Vectors

In the first series of experiments, four *A. tumefaciens* strains were compared, all based on the chromosomal background of C58: the octopine type GV2260 (McBride and Summerfelt, 1990), the nopaline type GV3101 (Holsters et al., 1980) and the succinamopine types EHA105 (Hood et al., 1993), and AGL-1 (Lazo et al., 1991). The transformation protocol was established by comparing the four strains all harboring the vector pCAMBIA1301^1^ (gene bank accession number: AF234297.1) carrying T-DNA consisting of the *hygromycin phosphotransferase* (*hpt*) gene under the control of the *CaMV35S* promoter and the *β-glucuronidase* (*gusA*, originally *uidA* of *Escherichia coli*, hereafter termed *gus*) gene with an intron of the *catalase* gene from *Ricinus communis* under control of the 35S promoter.

The vector pCAMBIA1380_DR5::GUS was obtained by cloning the *DR5::gus* sequence from the pGEM^®^-T-DR5::GUS plasmid (kindly provided by Günther Scherer, Ulmasov et al., 1997b) via restriction with *Eco*RI und *Sal*I into the plasmid pCAMBIA1380^1^ (gene bank accession number: AF234301.1). This vector was transformed into the *A. tumefaciens* strains EHA105, GV2260, and the TiAch5 strain LBA4404 (Hellens et al., 2000).

The plasmid with the integrated gene construct *roGFP2_Orp1* within the vector pH2GW7_c-roGFP2_LR_Orp1 (vector sequence: gene bank accession number FN398078.1, roGFP2: Hanson et al., 2004, yeast Orp1: gene ID: 854855, cloning strategy described in Scuffi et al., 2018) was kindly provided by Markus Schwarzländer (University of Bonn, now University of Münster). The plasmid used was 11187 bp in size and contained, in addition to the H_2_O_2_ sensing gene, the streptomycin-spectinomycin resistance gene *Sm/SpR* on the vector backbone and the *hpt* gene within the T-DNA; the *roGFP2_Orp1* and *hpt* genes were both under the control of the *CaMV35S* promoter (Schwarzländer et al., 2016). The vector was electroporated into the *A. tumefaciens* strains EHA105, GV2260, and LBA 4404.

### Hygromycin Sensitivity Test

Embryogenic cells were used as the target material for *A. tumefaciens*-mediated transformation. To identify concentrations suitable for the selection of transgenic cells, the sensitivity of the embryogenic callus to hygromycin and kanamycin was tested. Because even concentrations of 200 mgL^−1^ kanamycin could not consistently inhibit callus growth, we decided to use hygromycin as the selective agent in the transformation experiments. To identify suitable concentrations for the selection of transgenic cells, callus lines of the two *C. persicum* genotypes 56/2 and 3145 were grown on propagation medium containing 0, 5, 10, 15, or 20 mgL^−1^ hygromycin. Five replicates (6 cm Petri dishes) with 100 ± 10 mg embryogenic callus were prepared individually, and the whole experiment was repeated once. After 4 weeks, the callus fresh mass was recorded and expressed as a percentage of the initial mass plated.

### Transformation Protocol

#### Preparation of the *Agrobacterium tumefaciens* Culture

Bacterial glycerine stock cultures were regularly tested for the presence of the transformation vector by colony PCR, and only positive colonies were selected for inoculation in 10 mL YEB medium (5 gL^−1^ peptone, 5 gL^−1^ yeast extract, 5 gL^−1^ beef extract, 5 gL^−1^ sucrose, 0.493 gL^−1^ MgSO_4_, pH: 7.2) and the respective antibiotics (50 mgL^−1^ kanamycin; 50 mgL^−1^ rifampicin; for strain GV2260, 25 mgL^−1^ carbenicillin; for LBA4404, 10 mgL^−1^ streptomycin). After overnight culture at 28°C and 160 rpm, the suspensions were diluted 1:10 with an amendment of 50 mgL^−1^ acetosyringone after reaching an OD_600_ of 1.0. After the dilution reached an OD_600_ of 0.4–0.6, the bacteria were pelleted (3000 rpm, 10 min) and resuspended in liquid differentiation medium (see section “Regeneration of Plants via Somatic Embryogenesis”) at an OD_600_ of 0.5. This solution was supplemented with 50 mgL^−1^ acetosyringone and 0.03% Silwet^®^ L-77 and used for transformation.

#### Transformation of Embryogenic Cells

Three portions of 100 ± 10 mg embryogenic cells were plated in a thin layer in five to ten 6 cm Petri dishes containing 10 mL propagation medium (see section “Regeneration of Plants via Somatic Embryogenesis”), resulting in 15 to 30 replicates. These cells were subjected to a preculture of 10 days at 24°C in darkness. Thereafter, 300 μL of the transformation solution (see section “Preparation of the *Agrobacterium tumefaciens* Culture”) was pipetted on top of the cells of each replicate. After 2 days co-culture under the same culture conditions, the cells were washed by overlaying each replicate with 200 μl liquid propagation medium with 500 mgL^−1^ cefotaxime and pipetting this solution up and down several times. The cells of each replicate were then transferred to fresh propagation medium containing 500 mgL^−1^ cefotaxime in individual Petri dishes and cultured at 24°C in darkness for 10 days.

#### Selection of Transgenic Cells and Plant Regeneration

Ten days after co-culture, the cells were transferred to proliferation medium containing 500 mgL^−1^ cefotaxime and 5 mgL^−1^ hygromycin for selection. After 2 weeks, the cells were subcultured, and the selection pressure was increased to 10 mgL^−1^ hygromycin. Thereafter, every 4 weeks, the cultures were evaluated for growth and color and subcultured by plating them in thin layers. After three subcultures, cefotaxime was no longer added to the medium, because no outgrowth of bacteria was observed.

Starting from the second subculture after co-culture, cell proliferation of the putative transgenic cells was obvious. Thus, at the third subculture, parts of these actively growing cells were transferred to differentiation medium with 500 mgL^−1^ cefotaxime and 10 mgL^−1^ hygromycin, on which globular somatic embryos became visible after 3 weeks. After 4 to 5 weeks, torpedo stage embryos were singulated and further cultured on fresh medium of the same composition. Within 4 weeks, still in darkness at 24°C, most of the somatic embryos germinated and were then transferred to light (see section “Regeneration of Plants via Somatic Embryogenesis”) and to differentiation medium containing 5 mgL^−1^ hygromycin and 2.9 μM indole acetic acid (IAA) to promote root growth.

### Molecular Analyses of Transformed Regenerants

#### Extraction of Genomic DNA

DNA was extracted from callus, somatic embryos or leaf material using the NucleoSpin^®^ Plant II kit following the manufacturer’s instructions (Macherey-Nagel, Düren, Germany). The purified DNA was eluted in a volume of 100 μL elution buffer, and its concentration and purity were determined spectrophotometrically (Nanodrop, Peqlab, Erlangen, Germany).

#### Amplification of Transgenes via PCR

A multiplex PCR was used to amplify fragments of the *hpt* and *gus* genes. One 25 μL reaction contained 10 ng genomic DNA, 1 μL of each primer (Supplementary Table 1) at a concentration of 10 pmolμL^−1^, 0.75 μL dNTPs (10 mM), buffer (Williams et al., 1990), and 1U Taq polymerase (DNA Cloning Service, Hamburg, Germany). The protocol used for the multiplex PCR was as follows: initial denaturation at 94°C for 3 min; 15 cycles of 94°C for 45 s, 65°C for 45 s, 72°C for 70 s; 25 cycles of 94°C for 45 s, 58°C for 45 s, 72°C for 70 s; final elongation at 72°C for 7 min. To test for residual agrobacteria, PCR amplification of chromosomal DNA [*PicA* (Plant inducible chromosomal A) gene] was performed as described in Winkelmann et al. (2006b). PCR products were separated in a 1% agarose gel with 1 μgL^−1^ ethidium bromide for visualization of the bands at 300 nm.

For the transgenes *DR5::gus* and *roGFP2_Orp1*, separate PCRs were performed with the following thermocycler program: initial denaturation at 94°C, 3 min; 40/35 (for *DR5::gus* and *roGFP2_Orp1*, respectively) cycles of 94°C for 45 s, T_A_ (Supplementary Table 1) for 45 s, 72°C for 70 s for *DR5::gus* and for 45 s for *roGFP2_Orp1*; and finally 72°C for 7 min.

#### Southern Hybridization

Approximately 3 μg of genomic DNA from different transgenic plants and untransformed control plants was digested with 30 U *Hin*dIII overnight at 37°C before adding a further 10 U *Hin*dIII and incubating for 24 h. Following the report of Sriskandarajah et al. (2007), the DNA fragments were separated in 1% agarose gels and blotted onto a nylon membrane. The *hpt* probe, with a size of 578 bp, was digoxigenin-labeled using the DIG DNA Labeling Mix (Roche Applied Science Co., Mannheim, Germany) according to the manufacturer’s instructions. Plasmid DNA served as a positive control. All steps, including hybridization, washing and detection, were carried out as described in Sriskandarajah et al. (2007).

### GUS Assay

The histochemical GUS assay was carried out according to Jefferson (1987) for callus as well as somatic embryos and the leaf tissue of the regenerated plantlets. From 200 to 600 μL of the GUS staining buffer was placed into 2 mL reaction tubes containing the plant material. Infiltration of the tissue was promoted by evacuation at 200 mbar for 30–60 min. After incubation at 37°C for 16 h, the samples were bleached and fixed by immersion in increasing concentrations of ethanol (30–98%) before being evaluated under a stereomicroscope. Six weeks after co-culture, samples of all embryogenic callus replicates of the first series of transformation experiments were tested for GUS expression. *DR5::gus* transgenic somatic embryos were submitted to the GUS assay weekly after the transfer of embryogenic cells to differentiation medium.

### Statistical Analysis

The relative increase in callus fresh mass during the 4 weeks of culture in each hygromycin treatment was compared to that of the corresponding control. The data was log-transformed and statistically analyzed using the software R, version 3.4.2, by Dunnett’s test at *p* < 0.05.

## Results

### Selection Based on Hygromycin Sensitivity

**FIGURE 1:**
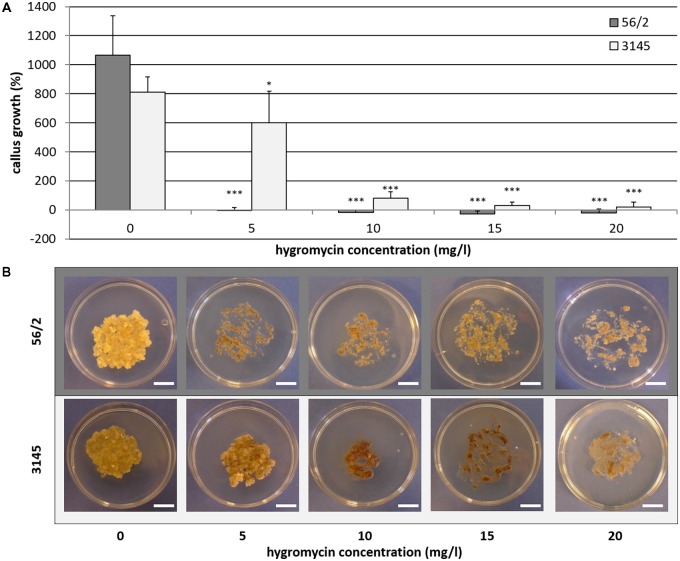
Genotype-dependent hygromycin sensitivity of *Cyclamen persicum*. **(A)** Callus growth as relative increase of fresh mass after 4 weeks on propagation medium, error bar = SD, asterisks indicate significant differences between the treatments and the corresponding control by Dunnett’s test (^∗^*p* ≤ 0.05 and ^∗∗∗^*p* ≤ 0.001), *n* = 5. **(B)** Callus cultures after 4 weeks on propagation medium under selective conditions, bars = 1 cm.

On the hygromycin-free control medium, embryogenic callus of the genotypes 56/2 and 3145 grew quickly, resulting in tenfold and eightfold fresh mass after 4 weeks of culture, respectively (**Figure 1**). For genotype 56/2, the lowest tested concentration of 5 mgL^−1^ hygromycin completely inhibited callus growth. The cells of genotype 3145 were much less sensitive and showed an increase in fresh mass of 600% on medium with 5 mgL^−1^ hygromycin (**Figure 1**); even at 10 mgL^−1^ hygromycin, cell proliferation occurred, although at a much lower level (82%) and with pronounced browning (**Figure 1**). Thus, for genotype 3145, selection should use 15–20 mgL^−1^ hygromycin, whereas for genotype 56/2, selection starting with 5 mgL^−1^ hygromycin (and after 2 weeks increasing to 10 mgL^−1^) is recommended.

### Comparison of Different *Agrobacterium tumefaciens* Strains

**FIGURE 2:**
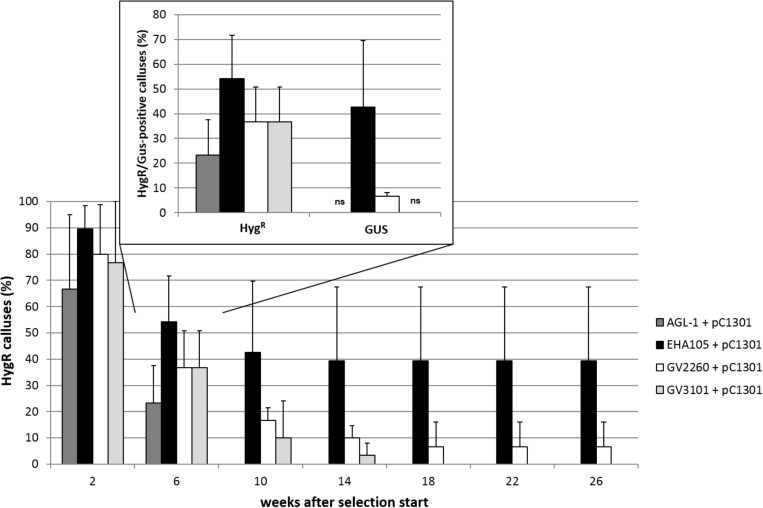
Transformation success for the different *A*. *tumefaciens* strains in terms of percentage of hygromycin-resistant (Hyg^R^) calluses over time for genotype 56/2. Inset shows percentages of Hyg^R^ calluses and GUS-positive (GUS) calluses 6 weeks after the start of selection. ns, no signal in GUS assay, pC1301, pCAMBIA1301. Replicate numbers: *n* = 30 for AGL-1, GV2260, and GV3101, *n* = 72 for EHA105. Whiskers indicate standard deviations between experiments.

**FIGURE 3:**
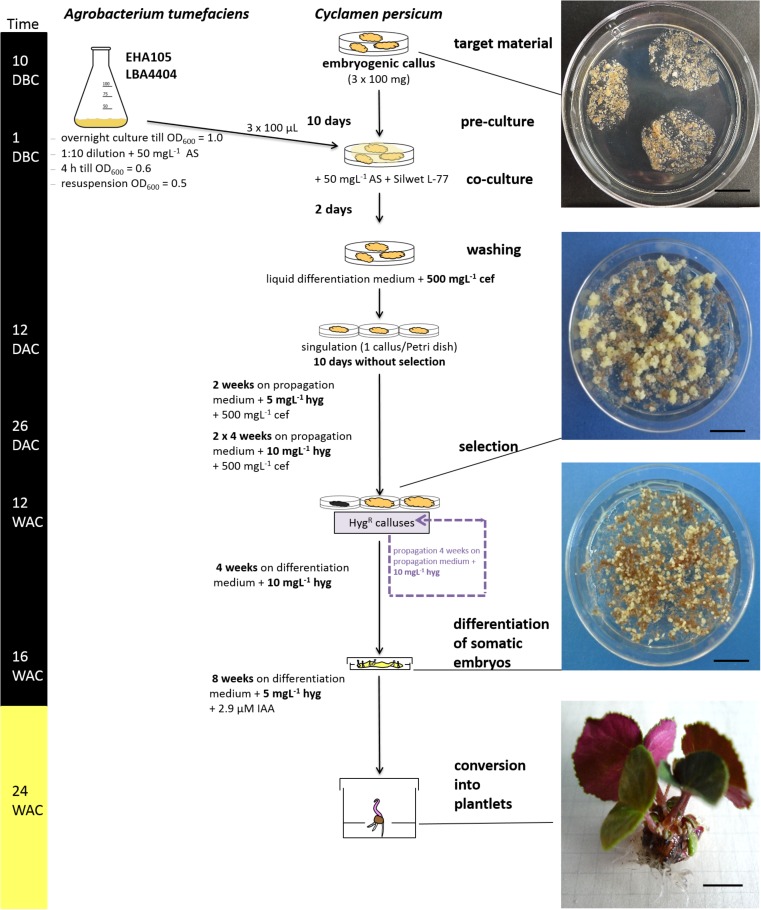
Overview of the validated transformation protocol for *C. persicum*. AS, acetosyringone; hyg, hygromycin; cef, cefotaxime; DBC, days before co-culture; DAC, days after co-culture; WAC, weeks after co-culture. Black bar = culture in darkness, yellow bar = culture under 16 h light. Size standards in photos represent 1 cm.

The aim of the first series of transformation experiments was to identify *A. tumefaciens* strains that are most efficient in transforming the embryogenic cells of *C. persicum*. In the first 2 weeks of selection, using a hygromycin concentration of 5 mgL^−1^, only a small percentage of calluses of genotype 56/2 stopped growing, and depending on the *A. tumefaciens* strain, between 67 and 89% of the calluses were transferred to fresh propagation medium with 10 mgL^−1^ hygromycin (**Figure 2**). After 4 weeks of culture at the higher hygromycin selection level, a clear reduction in viable calluses was recorded, although in strain EHA105, more than 50% of the calluses were still actively growing. At this time point, the first GUS assay revealed similar results for the different *A. tumefaciens* strains, although at a lower overall level (**Figure 2**, inlay). Interestingly, the GUS assay reflected the percentages of transformed calluses after 18 weeks very well. At the end of each subculture period, areas of actively growing cells among the dead brown cells were observed, and these living cells were then subcultured (**Figure 3**, photo selection). Further culture passages reduced the number of hygromycin-resistant and thus putatively transgenic calluses, until 18 weeks after start of selection, when a stable percentage was reached. The *A. tumefaciens* strain EHA105 resulted in the highest transformation rate of 42.6% followed by strain GV2260 with 6.7%, while no transformed calluses could be obtained with strains AGL-1 or GV3101 (**Figure 2**). The transformation rate for EHA105 was based on the highest replicate number, but from the standard deviation, the high variability between experiments becomes obvious. Although we tried to keep all handling of callus lines consistent, there is always variation among the Petri dishes from which callus was taken as starting material. This variation concerned callus color, consistency, embryogenesis, and growth rates. Lines with a high embryogenic potential were found to give rise to high transformation rates.

Ten weeks after the start of selection (= approximately 12 weeks after transformation), portions of actively growing embryogenic callus were plated on differentiation medium containing 10 mgL^−1^ hygromycin. Somatic embryos developed in high numbers within 4 weeks (**Figure 3**, photo differentiation), which were singulated and cultured for another 4 weeks on the same medium in Petri dishes before being transferred to larger vessels (250 mL) in light. Only 4% of the separated somatic embryos could be converted into plantlets on the differentiation medium with 10 mgL^−1^ hygromycin. However, from 1 g of embryogenic callus, 200 somatic embryos could be generated, on average. Root growth was inhibited on the hygromycin-containing medium compared to that of non-transformed control plants, but this effect was overcome by the addition of 2.9 μM IAA to the medium and reducing the hygromycin concentration to 5 mgL^−1^.

### Verification of Transgenic Plants

**FIGURE 4:**
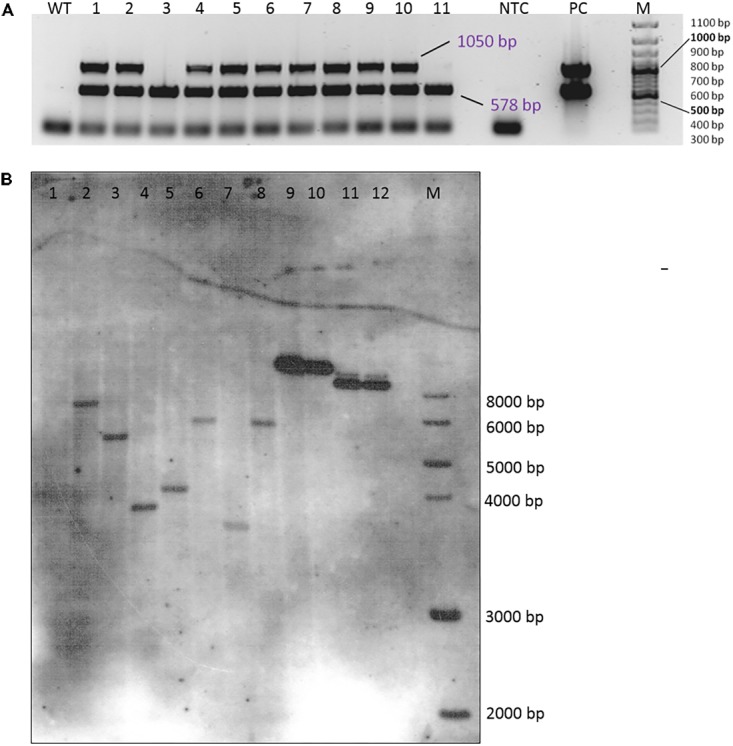
Verification of transgenic plants. **(A)** Multiplex PCR amplification of the *hpt* (578 bp) and the *gus* (1050 bp) gene fragments from DNA isolated from the leaves of 11 independent transgenic plants (genotype 56/2, EHA105 + pCAMBIA1301). WT, wild-type 56/2, DNA of a non-transformed plant; NTC, no template control (H_2_O); PC, EHA 105 + pCAMBIA1301; M = 100 bp size standard. **(B)** Southern hybridization of DNA from wild-type and transgenic plants of genotype 56/2 from different transformation experiments. The DNA was digested with *Hin*dIII and probed with a DIG-labeled *hpt* amplicon. 1 = WT, 2–3 = transformed with LBA4404 + pCAMBIA1380_DR5::GUS, 4: transformed with EHA105 + pCAMBIA1380_DR5::GUS, 5–8 = transformed with EHA105 + pCAMBIA1301, 9: 0.5 ng pCAMBIA1301, 10: 0.2 ng pCAMBIA1301, 11: 0.5 ng pCAMBIA1380_DR5::GUS, 12: 0.2 ng pCAMBIA1380_DR5::GUS, M: 1 kb DNA Ladder (New England Biolabs, Inc., Ipswich, MA, United States).

Integration of the transgenes was tested by a multiplex PCR allowing the simultaneous testing of the presence of both the *hpt* and *gus* genes (**Figure 4A**). In most plants that were regenerated from the transformation experiments, both genes were detected; only a few plants did not show a signal for the *gus* gene but had a clear band for the *hpt* gene (plants number 3 and 11 in **Figure 4A**). All DNA samples were also subjected to PCR to detect *A. tumefaciens* DNA and thus residual agrobacteria, but all were found to be negative in the *PicA* PCR (Supplementary Figure 1).

**FIGURE 5:**
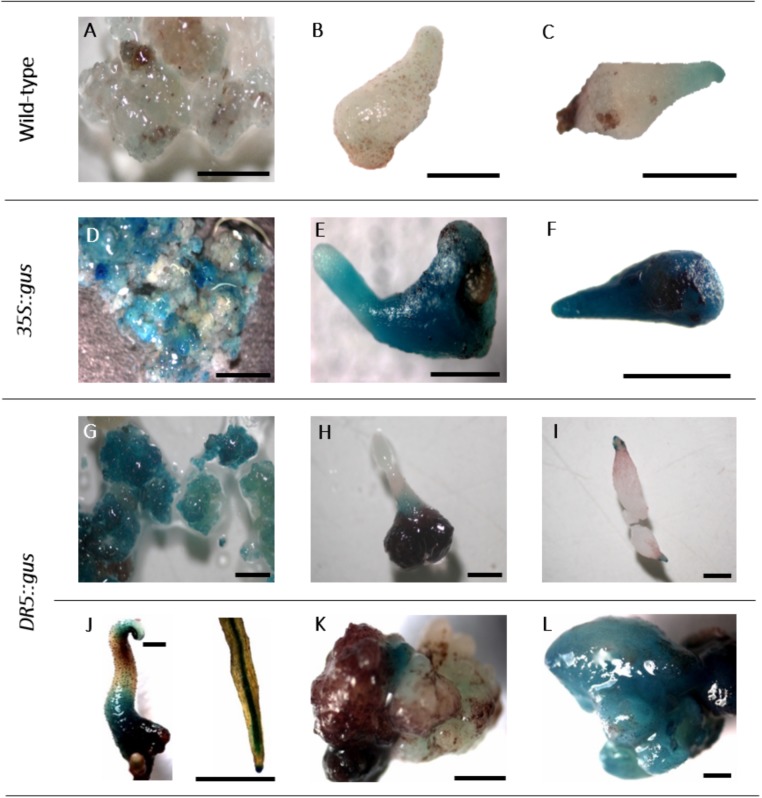
Histochemical GUS assay. **A–C**: non-transgenic lines of genotype 56/2: **A**: embryogenic callus, **B,C**: somatic embryos. **D–F**: *35S::gus* transgenic lines of genotype 56/2: **D**: embryogenic callus, **E,F**: somatic embryos. **G–L**: *DR5::gus* transgenic lines of genotype 56/2: **G**: embryogenic callus, **H,I**: somatic embryos, **J**: germinated somatic embryo and close-up of a root, **K,L**: malformed somatic embryos. Bars represent 1 mm.

The expression of the *gus* gene was proven in histochemical GUS assays. In callus cells sampled 6 weeks after the start of selection, GUS staining was already observed (**Figure 2**), and likewise, tests of somatic embryos and leaves revealed the deep blue coloration of the tissues, although to different extents (**Figures 5D–F**). The non-transgenic controls mostly did not show any blue color; however, in a few cells, a faint blue staining was observed (**Figures 5A–C**), which could be distinguished from the intense staining in the GUS-positive cells.

From the transformation experiments mentioned in the section “Comparison of Different *Agrobacterium tumefaciens* Strains,” 217 hygromycin-resistant plants were obtained overall, 38 of which were subjected to GUS assays and multiplex PCR (Supplementary Table 2). None of these 38 tested plants was found to test negative for all three parameters. Most of the plants (29, 76.3%) consistently tested positive for all traits. Four plants were found to contain *hpt* but lack the *gus* gene, and five plants gave contradictory results when the GUS assay was compared to the PCR amplification of the *gus* gene (Supplementary Table 2). Among these five plants, two were GUS positive but did not show positive signals in the *gus* PCR. Overall, these data indicated that the GUS assay gave valid results and that hygromycin selection was efficient.

A small subsample of four plants was submitted to a Southern hybridization analysis, which proved the integration of single copies of the *hpt* gene (**Figure 4B**, lanes 5–8). The four plants were obtained from calluses grown in different Petri dishes and from two different transformation experiments. Consequently, the different sizes of the hybridization signals indicate them to be independent transgenic events.

### *DR5::gus* Transgenic *C. persicum*

A first application of the transformation protocol was the transfer of the *gus* gene under the auxin-responsive promoter DR5. This set of transformation experiments included, in addition to the previously tested *A. tumefaciens* strains EHA105 and GV2260, the strain LBA4404 and consisted of three experimental replications with 10 Petri dishes each. At the end of the fourth culture passage after the start of selection, 50% of the calluses were actively growing on propagation medium with 10 mgL^−1^ hygromycin when transformed with EHA105 and LBA4404, whereas this percentage was much lower (26%) for strain GV2260. However, due to a longer first selection phase on 5 mgL^−1^ hygromycin (4 weeks instead of 2 weeks), the percentages of GUS-positive calluses better reflected the transformation efficiencies at this time point, which were 20% for LBA4404 and 7% for EHA105. Seventeen callus lines were randomly selected for molecular verification via PCR; nine of them were positive for the hpt gene, but only five were positive for the *gus* gene. Via Southern hybridization, three plants (two obtained from transformation with LBA4404 and one with EHA105) were shown to carry single copies of the *hpt* gene due to independent transgenic events (**Figure 4B**, lanes 2–4).

*DR5::gus* transgenic cells, somatic embryos and germinated embryos were analyzed in a histochemical GUS assay (**Figures 5G–L**). While the embryogenic cells were mostly stained intensely blue (**Figure 5G**), comparable to *35S::gus* transgenic lines (**Figure 5D**), in somatic embryos, GUS expression was mainly observed in the lower parts of the tubers and the root and the shoot poles (**Figures 5H,I**). This differential localization of the stain could be first observed 2 weeks after transfer to the differentiation medium. Germinated somatic embryos showed blue coloration in the tubers, the root tips, the root parenchyma, and later, the cotyledons (**Figure 5J**). Interestingly, the first analyses of malformed somatic embryos revealed that they either expressed only very low GUS activity or were completely stained blue (**Figures 5K,L**).

### *roGFP2_Orp1* Transgenic *C. persicum*

The aims of the third and final series of transformation experiments were (i) to test the transformation protocol with a second *C. persicum* genotype and (ii) to produce transgenic cyclamen carrying the redox sensor construct *roGFP2_Orp1*, which will enable hydrogen peroxide localization within living cells. Therefore, embryogenic callus of the genotypes 56/2 and 3145 was transformed following the established protocol, with the only alteration being doubled hygromycin concentrations for the selection of genotype 3145. The vector pH2GW7_c-roGFP2_LR_Orp1 was tested in the three *A. tumefaciens* strains EHA105, GV2260, and LBA4404. The protocol could be successfully applied in genotype 3145 (**Table 2**). All strains resulted in comparable transformation efficiencies of 3–5%, estimated as the percentage of hygromycin-resistant calluses 18 weeks after the start of selection. Transgenic plants were regenerated from the hygromycin-resistant calluses, and a first batch of 16 plants was tested for the presence of the transgenes by PCR. For all 16 plantlets, the bacterial gene *PicA* was not amplified, whereas all contained the *hpt* gene and all except one also carried the *roGFP2_Orp1* gene. Transformation efficiencies were much lower than those in the previous experiments (**Table 2**). Especially for genotype 56/2, only with the strain LBA4404 were three out of 180 calluses found to grow on selection medium after 18 weeks, corresponding to a transformation efficiency of only 1.7%. A first observation under the fluorescence microscope revealed the typical GFP fluorescence, which was strongest in meristematic cells (Supplementary Figure 2).

**Table 2:** Results of three transformation experiments to transfer the *roGFP2_Orp1* gene into the *C. persicum* genotypes 56/2 and 3145.

Genotype	*A. tumefaciens* strain	Number of calluses transformed	Hyg^R^ calluses 18 weeks after start of selection		No. of plants submitted to PCR	No. of plants testing positive by PCR for	
			Number	Percentage		*hpt*	*roGFP2_Orp1*
56/2	EHA105	60	0	0	0	–	–
	GV2260	30	0	0	0	–	–
	LBA4404	180	3	1.7	0	–	–
3145	EHA105	60	3	5.0	0	–	–
	GV2260	30	1	3.3	5	5	5
	LBA4404	75	4	5.3	11	11	10

*The experiments included a comparison of the three A. tumefaciens strains EHA105, GV2260, and LBA4404.*

## Discussion

### Hygromycin Sensitivity in *C. persicum* Is Genotype-Dependent

To select transgenic cells from among untransformed cells in embryogenic callus, hygromycin was chosen as a selective agent after trials with kanamycin had revealed a high tolerance in *C. persicum* embryogenic cells. This result is in agreement with those of earlier transformation studies that used seedlings as starting material and selected transgenic regenerants with 5–20 mgL^−1^ hygromycin (**Table 1**; Aida et al., 1999; Terakawa et al., 2008; Boase et al., 2010; Kai et al., 2012). The selection scheme established in this study started 10 days after co-culture with a concentration of 5 mgL^−1^, which was doubled after 4 weeks of culture. For the later phase of the root growth of transgenic plants *in vitro*, the concentration was again lowered to 5 mgL^−1^. Applying this selection scheme, transgenic plants were repeatedly generated, and thus, the balance was kept between stringent selection preventing chimeras and escapes on the one hand, and overly harsh selection resulting in rapid death of untransformed cells, which can also kill transformed cells due to their degradation products, on the other hand.

An interesting observation was the divergent sensitivity of the two genotypes under investigation (**Figure 1**). In consequence, it seems recommendable to define different selection schedules and concentrations for different *C. persicum* genotypes. For genotype 3145, a doubling of the hygromycin concentrations was employed in the transformation experiments with the *roGFP2_Orp1* gene. The reasons for the differences in sensitivity are unknown thus far but could involve uptake and detoxification mechanisms. In rice, Sultana et al. (2014) also reported genotypic differences in the reaction to hygromycin: the growth of embryogenic callus in three genotypes was inhibited at 40–47 mgL^−1^ hygromycin, whereas for germination of somatic embryos, even greater genotypic differences were observed for the inhibitory concentrations (32–62 mgL^−1^). This result confirms that genotypes and developmental phases differ in their hygromycin sensitivity and demonstrates variation between species.

### Transformation Efficiencies Depend on the *Agrobacterium tumefaciens* Strain

When preparing the *A. tumefaciens* solutions for genetic transformation experiments, it was observed that strain EHA105, in approximately 50% of the colonies tested from plated glycerine stocks, had lost the plasmid pCAMBIA1301. Thus, it was essential to prove the presence of the transformation vector in the starting material before preparing the transformation solution, by colony PCR.

The transformation solution was supplemented with 0.03% Silwet^®^ L-77, a detergent that is commonly used in the floral dip transformation protocol for *Arabidopsis thaliana* (Clough and Bent, 1998) but has also been shown to improve transformation efficiencies in wheat (Cheng et al., 1997).

In a series of four transformation experiments, the transformation efficiency, expressed as percentage of hygromycin-resistant calluses (**Figure 2**), varied greatly among the four *A. tumefaciens* strains used. The most efficient strain was EHA105 (42.6% transformation efficiency), followed by GV2260, while GV3101 and AGL-1 did not result in calluses growing in the presence of hygromycin 18 weeks after start of selection. EHA105 is a hypervirulent *A. tumefaciens* strain that also, in previous studies, was found to be suitable and efficient for the transformation of embryogenic cultures, for instance those of banana (Ganapathi et al., 2001), *Vitis vinifera* (Lopez-Perez et al., 2008), and *Citrus sinensis* (Dutt and Grosser, 2010). If previous work in *C. persicum* is considered (**Table 1**), the majority of studies successfully used strains EHA105 or LBA4404. A comparison of strains EHA105 and LBA4404 was included in the three transformation experiments transferring the *DR5::gus* gene. There, we found much lower transformation efficiencies overall, most likely due to insufficient selection pressures in weeks three and four (see section “*DR5::gus* Transgenic *C. persicum*”), but higher values of 20% for strain LBA4404. The differences between single experimental replications were most likely caused by uncontrollable physiological differences in the callus lines.

The transformation efficiencies (max. 42.6%) achieved in this series of experiments were high compared to those in most previous studies in *C. persicum* (**Table 1**): Aida et al. (1999) and Boase et al. (2002) reported 19 and 15.3% transformation efficiencies in the best variants, respectively. Only Tanaka et al. (2011) reached even higher values of 55% transformed explants.

### Confirmation of Transgenic Plants

Plants regenerated from the transformation experiments were analyzed by GUS assays and PCR for the presence of the *gus* and *hpt* genes. Residual agrobacteria were not detected in any of the plants, showing that the washing and thin plating on cefotaxime-containing medium were sufficient to fully suppress bacterial growth. Almost all regenerants (95%) contained the *hpt* gene, indicating successful selection. In addition, the results of the GUS assay and the *gus* PCR had high congruence (87%, Supplementary Table 2). Since the results of the GUS assay carried out 6 weeks after the start of selection resembled the final transformation efficiencies after 18 weeks (expressed as hygromycin-resistant calluses in **Figure 2**), a GUS assay allows an early estimation of the transformation efficiency. In four plants, only the *hpt* gene, but not the *gus* gene, was detected. On the T-DNA of the plasmid pCAMBIA1301, the *gus* gene is located close to the right border, where integration into the plant genome starts (Hellens et al., 2000). It is therefore not probable that these four plants were the result of incomplete incorporation of the T-DNA or deletions, which have been described to occur mainly at the left border (Li et al., 2007). Instead, the spectrophotometric measurements of the DNA extracts of these four plants revealed low absorption ratios (A_230_/A_260_), and thus, these extracts were contaminated with phenols and/or polysaccharides. One explanation for the missing band for the *gus* gene could be a higher sensitivity of the *gus* primers to these impurities.

By Southern hybridization, it was proven that single copies of the transgene (in this case *hpt*) were integrated (**Figure 4B**) and that different plants represented independent transgenic events. Further analyses of the other transgenes (*gus* and *DR5::gus*) and of a higher number of plants will allow the estimation of frequencies of single copy events for the genes of interest.

The histochemical GUS assay allowed early identification of transformed cells and clearly indicated the typical deep blue coloration in callus cells (**Figure 5D**). However, in a few samples of non-transformed callus cells as well as somatic embryos, a faint but clear blue tone was also observed (**Figures 5A,C**). Residual agrobacteria as the cause of these blue shades can be excluded, because the intron-containing *gus* gene version was used, which only be spliced by eukaryotic cells. Moreover, the *PicA* PCR was negative in all tested DNA samples. According to Jefferson (1987), blue background signals can occur when long incubation times are chosen, as in the present study. Furthermore, intrinsic GUS-like activities have been reported in other plants previously (Hu et al., 1990; Taylor, 1997). However, the faint coloration of the controls allowed them to be distinguished from the deep blue color and sharp localization of transgenic GUS-expressing cells.

When callus cells were subjected to GUS assays, they turned out to be mixtures of GUS-positive and GUS-negative cells, even within one cell aggregate (**Figure 5D**). This result can be explained by either a chimeric structure of the callus lines, including transgenic cells and escapes; an inhomogeneous infiltration of the GUS staining solution; or low expression levels of the *gus* gene in some cells. In principle, the 35S promoter controlling *gus* gene expression seemed to be active in *C. persicum* callus cells (**Figure 5D**), somatic embryos (**Figures 5E,F**) and the young leaves of regenerated plants (not shown).

### *DR5::gus* Transgenic Cyclamen Allow Visualization of Auxin Gradients

Transgenic cyclamen plants were generated carrying the *DR5::gus* construct. From the auxin-responsive element DR5, which was found naturally in the promoters of auxin-responsive genes, Ulmasov et al. (1997a, b) developed the synthetic DR5 promoter that was used in our study. They combined seven repeats of the DR5 motif with a fragment of the *Cauliflower mosaic virus* 35S promoter and the translation-enhancing omega sequence of *Tobacco mosaic virus*. In the *DR5::gus* construct, this synthetic promoter, being efficiently activated by auxin, controls the expression of the *gus* gene in order to visualize auxin in tissues. The *DR5::gus* gene has been used in a wide range of plant species to visualize auxin distribution in different organs and different developmental processes, for instance *A. thaliana* (Kurczynska et al., 2007), *Pisum sativum* (DeMason and Polowick, 2009), poplar (Chen et al., 2013), or strawberry (Estrada-Johnson et al., 2017). Moreover, it has become a widely used tool in fundamental research of auxin homeostasis and signaling (e.g., Deng et al., 2016; Lin et al., 2016). In *DR5::gus* transgenic *A. thaliana*, Kurczynska et al. (2007) observed overall high auxin concentrations in globular somatic embryos, but a differentiation in embryos in the heart stage, with elevated concentrations in the protoderm and the root and shoot poles. Cotyledonary somatic embryos expressed GUS activity mainly in the roots, vascular tissue, and parenchyma cells of the hypocotyl (Kurczynska et al., 2007). Using the *DR5::gus* approach and comparing somatic to zygotic *A. thaliana* embryos, Bassuner et al. (2007) found striking similarities in the mature embryos of both types. Both accumulated auxin in the tips of the roots and cotyledons. Interestingly, malformed *A. thaliana* somatic embryos often lacked the auxin signals detected by DR5::gus in their roots (Nowak et al., 2012).

For *C. persicum*, we performed the first analyses of the *DR5::gus* transgenic somatic embryos and could observe a more or less uniform blue coloration during the first week of differentiation, until the globular stage. Beginning in the second week, an auxin gradient, with higher GUS expression first in the root pole and later at the tip of the developing cotyledon, was seen (**Figures 5H,I**). Comparable to *A. thaliana*, in later stages, the root tips and root parenchyma cells, as well as the hypocotyl-derived tubers, showed blue signals. Malformed somatic embryos were either lacking GUS activity or were more or less fully stained (**Figures 5K,L**).

An auxin gradient is needed to establish polarity and pattern formation in early embryogenesis (Möller and Weijers, 2009). Since somatic embryogenesis protocols often involve high auxin concentrations in the induction phase, the establishment of an auxin gradient is based on controlled efflux as well as polar auxin transport, as discussed for Norway spruce somatic embryos (Hakman et al., 2009). Future studies in *C. persicum* could focus on more detailed time-line analyses of auxin distribution in *DR5::gus* transgenic lines, a comparison of somatic and zygotic embryos and the response of auxin distribution to exogenously applied plant growth regulators. *DR5::gfp* transgenic plants would help to overcome the limitations of the histochemical GUS assay and would allow auxin localization in living cells.

Although the GUS staining pattern reflected the expected auxin distribution well, a proof for the functionality, i.e., auxin sensitivity of the *DR5::gus* construct in *C. persicum* still is required. First trials to achieve this evidence were undertaken by incubating *DR5::gus* transgenic *C. persicum* roots in naphthalene acetic acid (NAA) at 50 μM for 24 h, as also used in previous studies (Ulmasov et al., 1997b; Estrada-Johnson et al., 2017). The GUS staining was observed in root tips and vascular tissue, but was not different from the water incubated control roots. However, in petioles, a deeper blue staining of the vasculature and blue coloration at the cut surfaces was observed after an incubation at 100 μM NAA for 2 h (Supplementary Figure 3). Thus, like previously reported for *DR5::gus* transgenic poplar (Chen et al., 2013), it will be obviously necessary to identify the appropriate type of auxin, its concentration and the duration of application in a organ-specific way in order to demonstrate the response of the promoter GUS construct in *C. persicum*.

### *roGFP2_Orp1* Transgenic Cyclamen as a Basis for ROS Localization

In the process of somatic embryogenesis, stress plays an important role (Karami and Saidi, 2010). Upon stress, plants produce ROS, which have a dual role in stress response and development. In high concentrations, ROS are toxic and will cause cell death, whereas in low concentrations, they act as important signal molecules for developmental processes (Mittler et al., 2011; Kocsy et al., 2013). Furthermore, the ROS signaling pathway is linked to plant hormone signaling, as shown in cotton somatic embryogenesis, where auxin signaling was shown to be regulated by ROS homeostasis (Zhou et al., 2016). Due to rapidly acting detoxification systems, ROS detection is not trivial, and fluorescent protein redox sensors such as roGFP2_Orp1 offer the great advantage of localization of ROS, in this case H_2_O_2_, in living cells (Schwarzländer et al., 2016). The emitted light wavelengths of the roGFP2 protein differ between the oxidized and reduced state and thus allow ratiometric imaging using confocal laser scanning microscopy (Schwarzländer et al., 2016).

To better understand the role of ROS in the process of somatic embryogenesis in comparison to zygotic embryogenesis, the redox sensor gene *roGFP2_Orp1* was transferred to *C. persicum* in this study. By PCR, it could be shown that the *roGFP2_Orp1* gene was present in plantlets regenerated from transformation experiments of genotype 3145 with *A. tumefaciens* strains GV2260 and LBA4404 (**Table 2**). Thereby, it was shown that the established transformation protocol could be successfully transferred to another genotype of a distinct gene pool and to another vector system. Differentiation of additional plants from the hygromycin-resistant calluses is in progress. All 16 regenerated plantlets were positive by PCR for the *hpt* gene, and only one plant was negative in the PCR testing for the *roGFP2_Orp1* gene. Again, the DNA of the negative sample (for roGFP2_Orp1) had a low A230/A260 ratio when analyzed spectrophotometrically, pointing to a possible inhibition of the PCR by polyphenols or polysaccharides. The reasons for the low transformation efficiencies most likely can be found in the different vector system, the larger size of the transferred DNA and the status of the plant material. Under fluorescent light, the functionality of the roGFP2 protein was proven, but testing the functionality of the redox sensing awaits labor-intensive studies under a confocal laser scanning microscope.

## Conclusion

An efficient transformation protocol for *C. persicum* starting from embryogenic callus has been established and successfully used to transfer genes of interest into different genotypes. This protocol does not depend on seedlings, which have genetic variability and the value of which has not been tested when used for transformation. Instead it is based on the regeneration via somatic embryogenesis starting from explants of adult plants which is highly efficient and connected to low percentages of aberrations in some genotypes (Schwenkel and Winkelmann, 1998). Embryogenic callus can be induced in the vast majority of genotypes (Winkelmann and Serek, 2005), although in different percentages. Additionally, the maintenance of embryogenic cultures differs between genotypes, with approximately 10–20% of the genotypes being easy to maintain. This fact may limit the applicability of the established transformation system for commercial breeders, if transgenic cyclamen would be accepted by the public. However, our intention is to use this transformation system for gene functional analyses, hence a set of two to three genotypes with different genetic backgrounds that are stably propagated as embryogenic cultures is sufficient. For success in transformation, we regard the following factors as important: highly embryogenic cell lines, the use of Silwet^®^ L-77 as a surfactant during co-culture, an appropriate selection schedule with hygromycin, and *A. tumefaciens* strains EHA105 and LBA4404. Because somatic embryos develop from few or even single cells within embryogenic callus cultures, we assume a low risk of chimeric regenerants, but this must be proven in further studies.

Applying the protocol established here, if a lower efficiency of 10% is taken as a basis for calculation, 100 callus portions of 100 mg would be needed to generate 10 independent transgenic lines. After 14 weeks (**Figure 3**) on selection medium (2 weeks with 5 mgL^−1^ hygromycin and two passages of 4 weeks with 10 mgL^−1^ hygromycin), the surviving embryogenic callus lines can be plated on differentiation medium. After 4 weeks, 50 somatic embryos should be picked per line to obtain two transgenic plants per event, on average.

## Author Contributions

SR performed major parts of the experimental work and data analysis and revised the manuscript. SM and AK performed parts of the experimental work and data analysis and revised the manuscript. MB conceived and designed the experiments and the manuscript structure. TW contributed to the experimental design and wrote the manuscript.

## Conflict of Interest Statement

The authors declare that the research was conducted in the absence of any commercial or financial relationships that could be construed as a potential conflict of interest.

## Footnotes

1 www.cambia.org
