# Supplementary material for: Embryogenic Callus as Target for Efficient Transformation of Cyclamen persicum Enabling Gene Function Studies
Source: Front Plant Sci. 2018 Jul 24;9:1035. doi: 10.3389/fpls.2018.01035 (PMC6066641; doi:10.3389/fpls.2018.01035)
Supplement: Supplementary file 1 [file Table_1.docx]

**Embryogenic callus as target for efficient transformation of *Cyclamen persicum* enabling gene function studies**

**Svenja Ratjens, Samuel Mortensen, Antje Kumpf, Melanie Bartsch, Traud Winkelmann**

Institute of Horticultural Production Systems, Leibniz Universität Hannover, Hannover, Germany

*** Correspondence:** Traud Winkelmann, Institute of Horticultural Production Systems, Leibniz Universität Hannover, Herrenhaeuser Str. 2, D-30419 Hannover, Germany.

Traud.winkelmann@zier.uni-hannover.de

**Supplementary Material**

**Supplementary Table 1:** Primer sequences, annealing temperatures and sizes of amplified fragments

| **Target gene** | **Primer name** | **Sequence (5ˈ🡪 3ˈ)** | **Annealing**  **temperature [°C]** | **Size of PCR product [bp]** |
| --- | --- | --- | --- | --- |
| ***PicA*** | PicA1  PicA2 | ATGCGCATGAGGCTCGTCTTCGAG  GACGCAACGCATCCTCGATCAGCT | 63 | 550 |
| ***gus*** | GUS1_pCambia  GUS2_pCambia | GATCAGCGTTGGTGGGAAAG  CCTGTAAGTGCGCTTGCTGAG | 65 | 1080 |
| ***hpt*** | hpt_s  hpt_as | GATGTTGGCGACCTCGTATT  GATGTAGGAGGGCGTGGATA | 58 | 578 |
| ***DR5::gus*** | DR5-GUS-for | TCC CTT TTG TCT CCC TTT TG | 50.7 | 393 |
|  | DR5-GUS-rev | AAT ATC TGC ATC GGC GAA CT |  |  |
| ***roGFP2_Orp1*** | GFP2_Orp_fw | ACGTAAACGGCCACAAGTTC | 58 | 187 |
|  | GFP2_Orp_rev | AAGTCGTGCTGCTTCATGTG |  |  |

**Supplementary Table 2:** Overview of the results of different methods of verification for transgenic plants obtained from the transformation experiments involving genotype 56/2 and different strains of *Agrobacterium tumefaciens.* Regenerated plants were subjected to a GUS assay and a multiplex PCR to prove the presence of the *hpt* and *gus* genes.

| Group | Verification method | | | Number of plants | Percentage [%] |
| --- | --- | --- | --- | --- | --- |
|  | GUS assay | *hpt* (PCR) | *gus* (PCR) |  |  |
| 1 | + | + | + | 29 | 76.3 |
| 2 | + | + | - | 2 | 5.3 |
| 3 | + | - | - | 2 | 5.3 |
| 4 | - | + | + | 3 | 7.9 |
| 5 | - | + | - | 2 | 5.3 |
| 6 | - | - | - | 0 | 0 |

1 2 3 4 5 NTC PC M


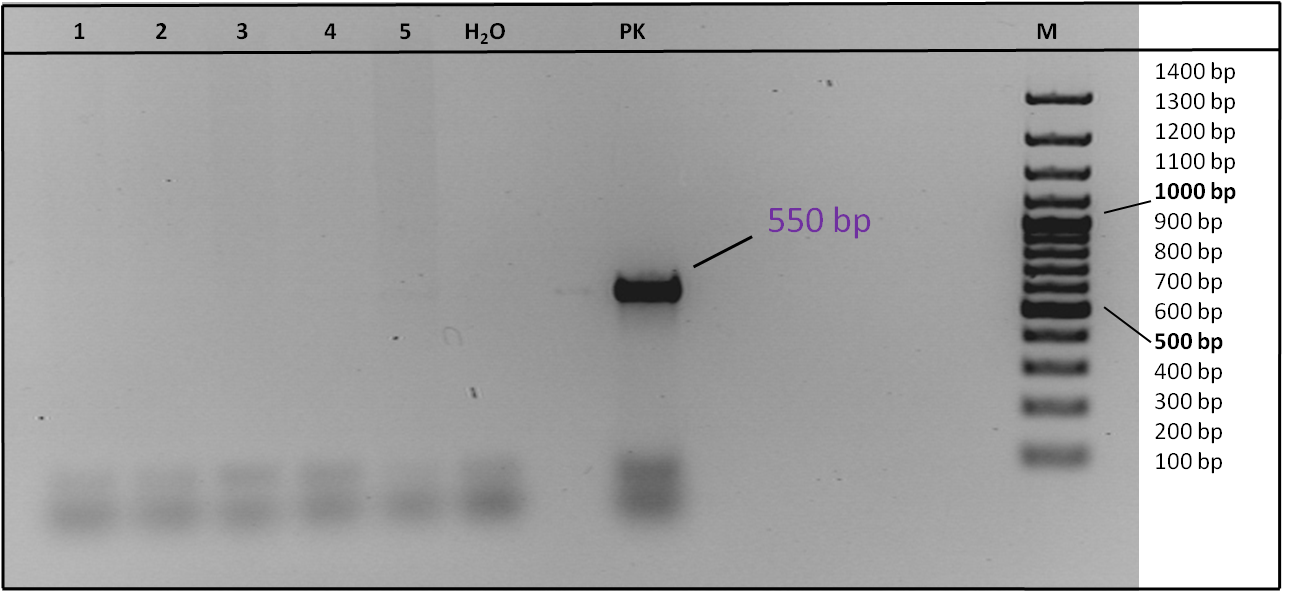


**Supplementary Figure 1:** PCR amplification of a fragment of the *PicA* gene to test for residual agrobacteria. 1-5 = transgenic lines of genotype 56/2 obtained from two independent transformation experiments with EHA 105 + pCAMBIA1301. NTC = no template control (H_2_O), PC = EHA 105 + pCAMBIA1301, M = 100 bp size standard.


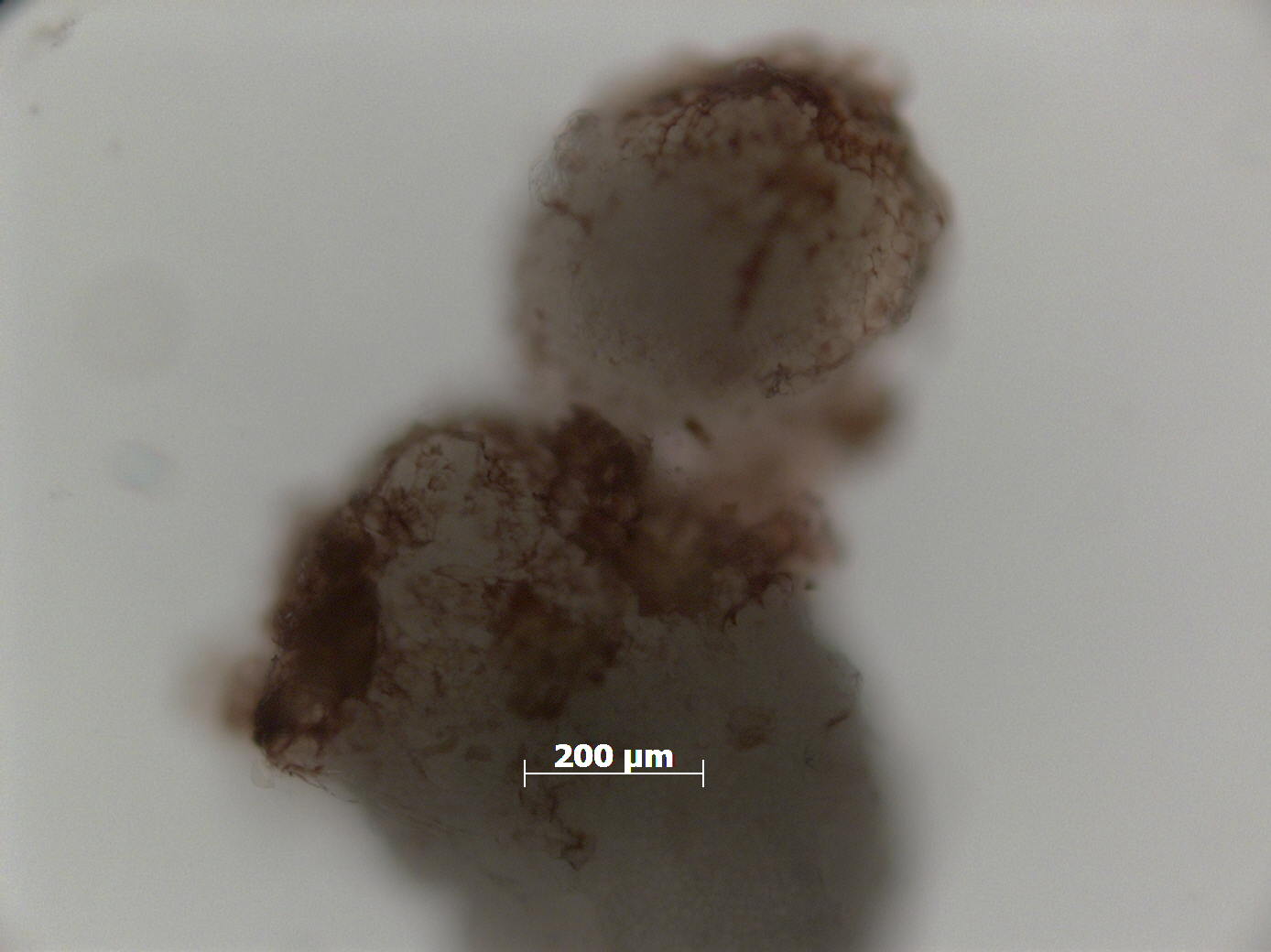

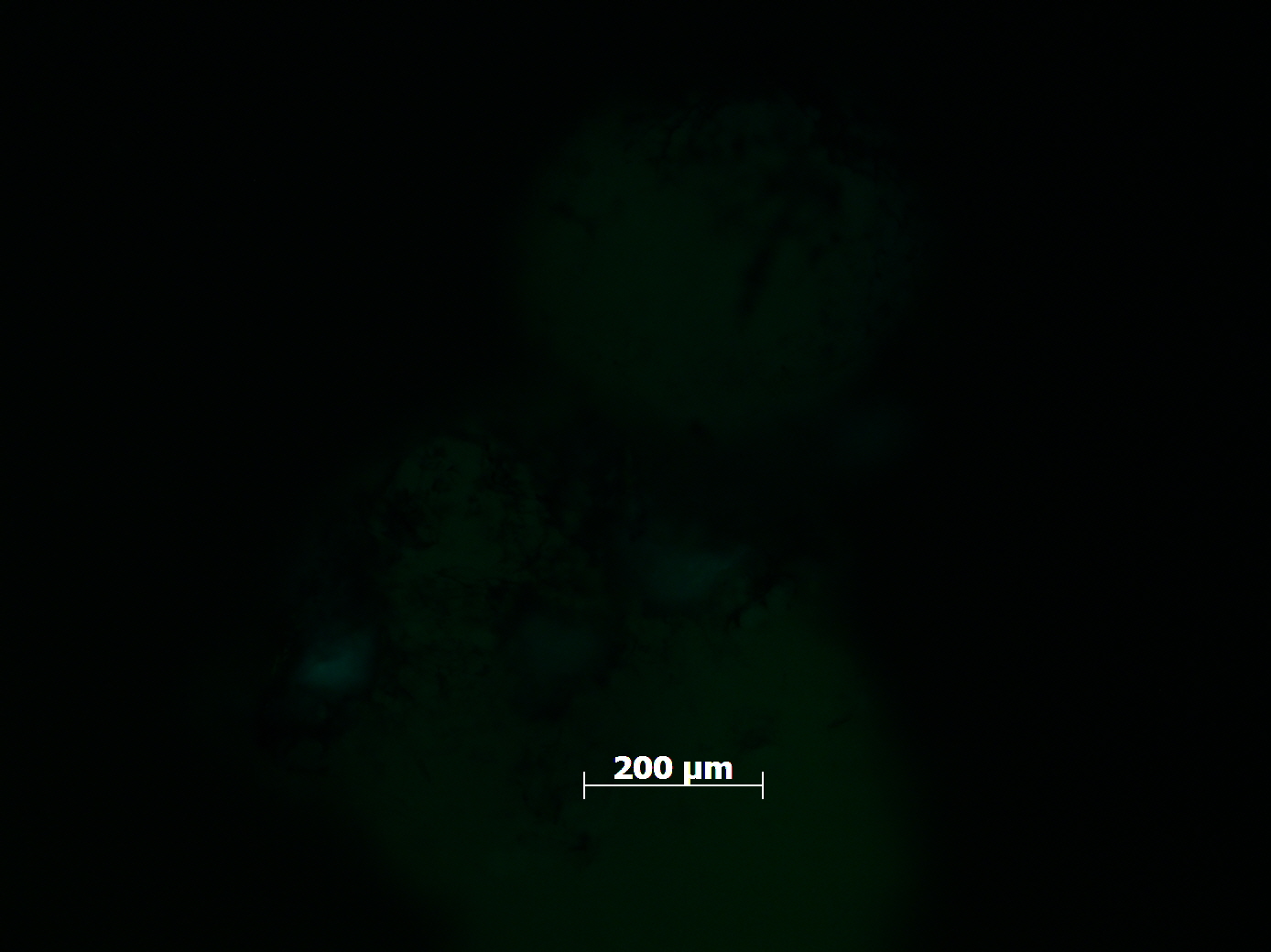

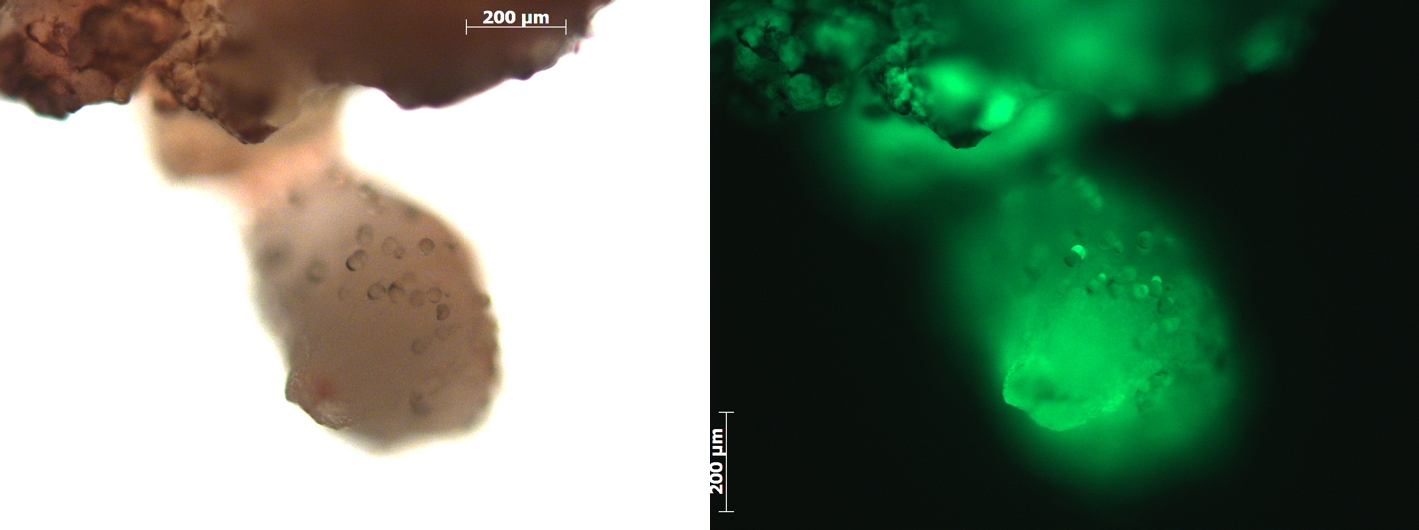


**A**

**C**

**B**

**D**

**Supplementary Figure 2:** Wild-type (A, B) and *roGFP2_Orp1* transgenic somatic embryo of genotype 3145 transformed with the *A. tumefaciens* strain LBA4404 (C,D) under visual light (A,C) and fluorescent light (B, D). Photographs were taken under the microscope Zeiss Axioscope 2 using the software Zeiss Axiovision and filter set 38 (excitation BP470/40, beam splitter FT495, emission BP525/50).


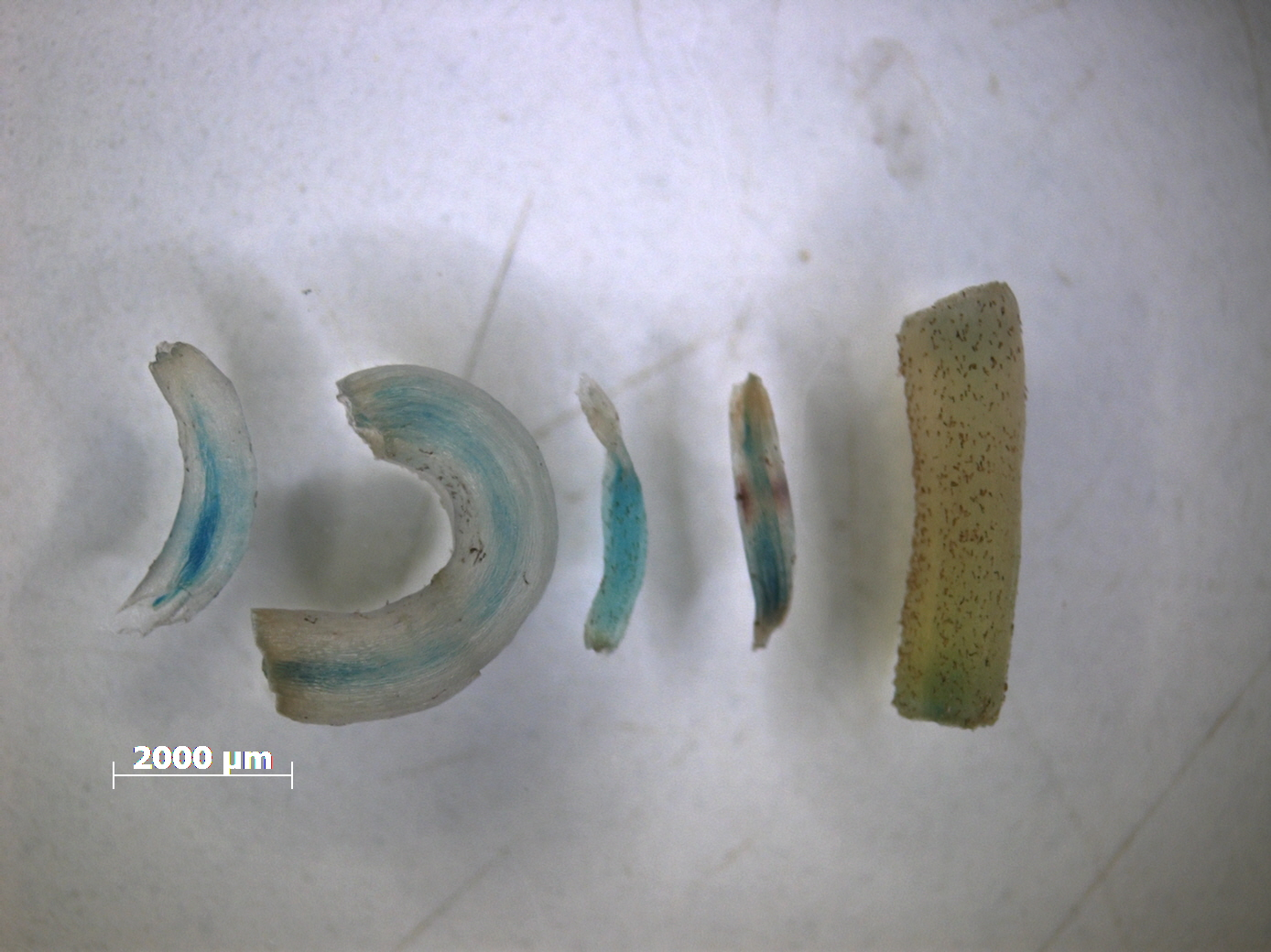


**A**


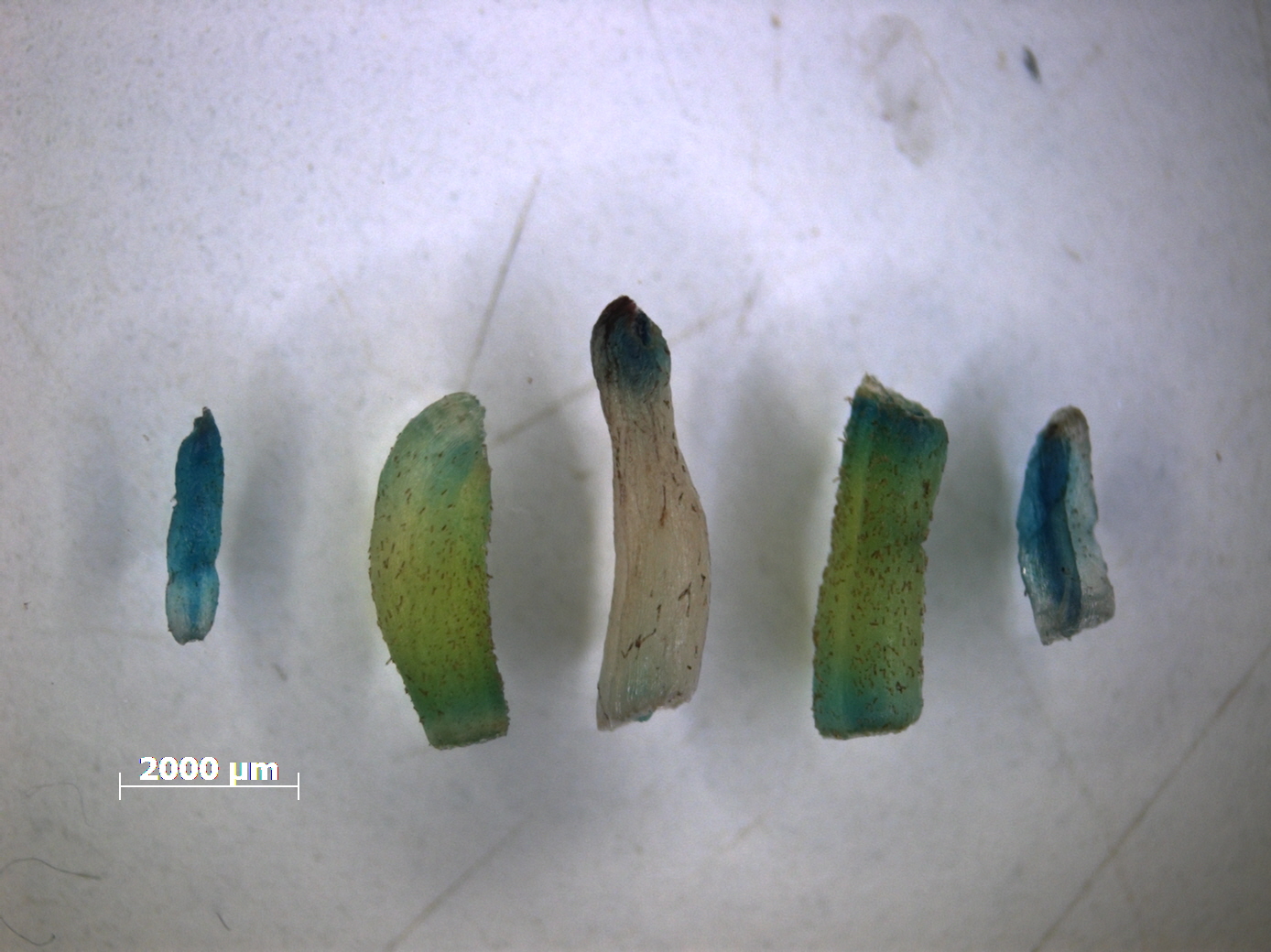


**B**

**BB**

**Supplementary Figure 3:** Histochemical GUS assay of petiole segments of *DR5::gus* transgenic in vitro plantlets of genotype 3145 after a 2 h incubation in H_2_O (A) or 100 µM NAA (B).
